# Differentiation of acute versus chronic skin rejection in a rodent model of vascularized composite allotransplantation

**DOI:** 10.3389/fimmu.2025.1672754

**Published:** 2025-09-30

**Authors:** Julia Thomé, Maike Lind, Maxime Schmitt, Laura Schneider, Jurij Kiefer, Rebecca Schäfer, Emma Freise, Thierry Christmann, Sheena Kreuzaler, Branislav Kollár, Steffen U. Eisenhardt

**Affiliations:** ^1^ Department of Plastic and Hand Surgery, Medical Center and Faculty of Medicine, University of Freiburg, Freiburg, Germany; ^2^ Institute of Pathology, University Hospital Giessen-Marburg (UKGM), Site Marburg – Philipps-University Marburg, Medical Faculty of the University of Marburg, Marburg, Germany; ^3^ Orlando Health Aesthetic & Reconstructive Surgery Institute, Orlando, FL, United States

**Keywords:** vascularized composite allografts, allograft rejection, acute rejection, chronic rejection, Banff criteria, rodent VCA model

## Abstract

**Background:**

Vascularized composite allografts (VCA) have evolved to be a potential option in complex reconstructive surgery. However, managing acute and chronic allograft rejection remains challenging. This study underlines differences between acute and chronic rejection in skin based on Banff criteria using rodent VCA models, enhancing comprehension of the underlying immunopathology.

**Methods:**

We compared whole tissue sections of fresh frozen skin from a rat hindlimb allograft transplantation model of acute and chronic rejection, respectively (n=7), stained with Hematoxylin Eosin-, Periodic Acid Schiff- and Masson’s Trichrome. Assessment followed the Banff 2007 working classification of skin-containing composite tissue allograft pathology, also considering the Banff 2022 VCA Working Group’s consensus. Immune cell infiltration was further analyzed via immunofluorescence.

**Results:**

Histopathological criteria effectively distinguished both acute and chronic rejection from healthy control skin. However, substantial overlap was observed, including perivascular infiltrates. Chronic rejection presented distinct features such as band-like lymphohistiocytic infiltrates, loss of rete ridges and adnexal structures, fibrosis, vasculitis, and allograft vasculopathy. Immune cell infiltration increased in both rejection groups.

**Conclusion:**

This study validates the application of the updated Banff classification in rat VCA rejection models, highlighting overlapping and distinct features of acute and chronic rejection patterns. Clear differentiation between acute and chronic rejection remains challenging, as no single criterion provides absolute diagnostic certainty and multiple pathways with transitional forms are involved. In our cohort, allograft vasculopathy, loss of rete ridges, and band-like lymphohistiocytic infiltrates were the most distinctive features, underscoring the need for an integrative diagnostic approach. The findings reflect patterns seen in human chronic active rejection and underscore the need for further research to better understand the mechanisms driving sustained inflammation and tissue remodeling in VCA rejection.

## 1 Introduction

Vascularized composite allografts (VCA) are utilized in complex reconstructive surgery, as for burn victims or after serious trauma. These allogeneic transplants comprise various tissue types, including blood vessels, skin, nerves, muscles, tendons, and in some cases bones. Since the first allogeneic transplantation of a human hand in 1998 (1), there has been an expansion in application fields, with overall more than 150 VCAs performed worldwide, including at least 50 facial transplants (2), as well as penile and abdominal wall transplants (3–5). Despite the differences herein, they all share the common characteristic of being composed, at least in part, of skin. Furthermore, they are all underlying unique immunological mechanisms in respect to acute (AR) and chronic rejection (CR). However, as a part of the immune system itself, the skin often is the key location in those rejection processes (6–9).

Compared to solid organ transplants, rejection happens far more often in VCA, though antibody-mediated rejection (ABMR) is rare (10–13). Instead, the skin is more commonly affected by cell-mediated rejection (14). For this reason, the roles of various immune cell types, including mast cells (15), T cells (16), granulocytes and monocytes (17) are pivotal in understanding the immunopathology of skin rejection and its corresponding inflammation. Recently, IL-17A–producing T cells and classical monocytes have been reported to be associated with a rapid immune response in the rejection of VCAs (18). Furthermore, the heterogeneity of VCAs continues to present a major challenge. While hand transplantations consistently involve bone, facial transplantations frequently include mucosal tissue. Since these tissue types exhibit distinct immunogenic properties, they can elicit different rejection responses. Recent studies have identified B-cell infiltration as a novel rejection pathway specifically in mucosal rejection (19). Biopsies obtained from mucosal tissue have been reported to demonstrate higher sensitivity for the diagnosis of rejection compared with conventional skin biopsies (20). Furthermore, advanced skin rejection has not been observed in the absence of concomitant mucosal rejection (21). This suggests that surface epithelia are particularly affected by rejection; at the same time, they provide convenient access for biopsy sampling. Nevertheless, skin biopsies are still the main tool in diagnosis of VCA rejection in clinical routine (22, 23). Moreover, the skin is exposed to external forces and environmental influences, at the same time allowing for macroscopic non-invasive examination and monitoring (7, 23). These unique characteristics not only make the skin a critical target of rejection but also provide a valuable system for developing and validating methods to assess alloimmune responses.

The Banff classification, a pivotal framework in evaluating allograft rejection, has historically shaped our comprehension of skin-containing composite tissue allograft pathology in clinical contexts (23). Pioneering works delineate the foundation upon which the Banff classification system was built and underscore the significance of characterizing skin rejection (23, 24). Though, until recently, it was lacking specific criteria for CR (25).

CR is often considered the result of repeated acute rejection episodes (26). Nevertheless, it remains unclear which additional features distinctly separate AR from CR and clarify diagnosis. AR often presents with perivascular lymphocytic infiltrates and epidermal involvement with inflammatory reactive epithelial changes (27), whereas CR is primarily characterized by a more vasculopathic appearance and fibrotic changes of dermal stroma (10, 22, 26). Though, the distinctive features still appear difficult to define, as a recently published long-term analysis of facial transplantations reported chronic rejection in 40% of patients without involvement of allograft vasculopathy (28). Since the majority of VCA experimental research is conducted using rat models (29), we used such models of AR and CR to examine this issue. Our aim was to validate the updated Banff classification system, which is applied in clinical practice to diagnose rejection in human VCAs. By comparing acute and chronic rejection, we further sought to elucidate the distinctions between the respective diagnostic criteria, thereby contributing to the refinement of histopathological evaluation methods in VCAs.

## 2 Methods

### 2.1 Animal model

For investigation of AR (n=7), we used a *major mismatch* model with Lewis rats as donors and seven Brown Norway (BN) rats as hindlimb allograft recipients as described previously (30, 31). No immunosuppression was applied for AR to occur. For CR (n=7) we used a *minor mismatch* model with seven Wistar Kyoto (WK) rats as hindlimb allograft recipients experiencing multiple episodes of AR before transitioning into CR. Surgical procedure was conducted in a similar fashion in both groups. In CR, all animals were checked daily for signs of clinical rejection over the 90-day course of the experiment. In case of mild rejection signs (swelling, redness, edema) 10mg/kg bodyweight Ciclosporin A and 2mg/kg bodyweight Dexamethason were applied intraperitoneally. This procedure was repeated daily until all signs of acute rejection faded. On average the animals experienced 4–5 rejection episodes and showed clinical signs of chronic skin rejection 30 days prior to the endpoint of the experiment. The respective healthy leg was used as a control, underlying systemic effects of rejection.

All animals were housed under standard conditions in the animal facility of the medical center of the University of Freiburg. All experiments were conducted according to the ethical policies and procedures and were approved by the ethics committee at the University of Freiburg, Germany (No. 35-9185.81/G-16/53 and G-21/102).

**Figure 1:**
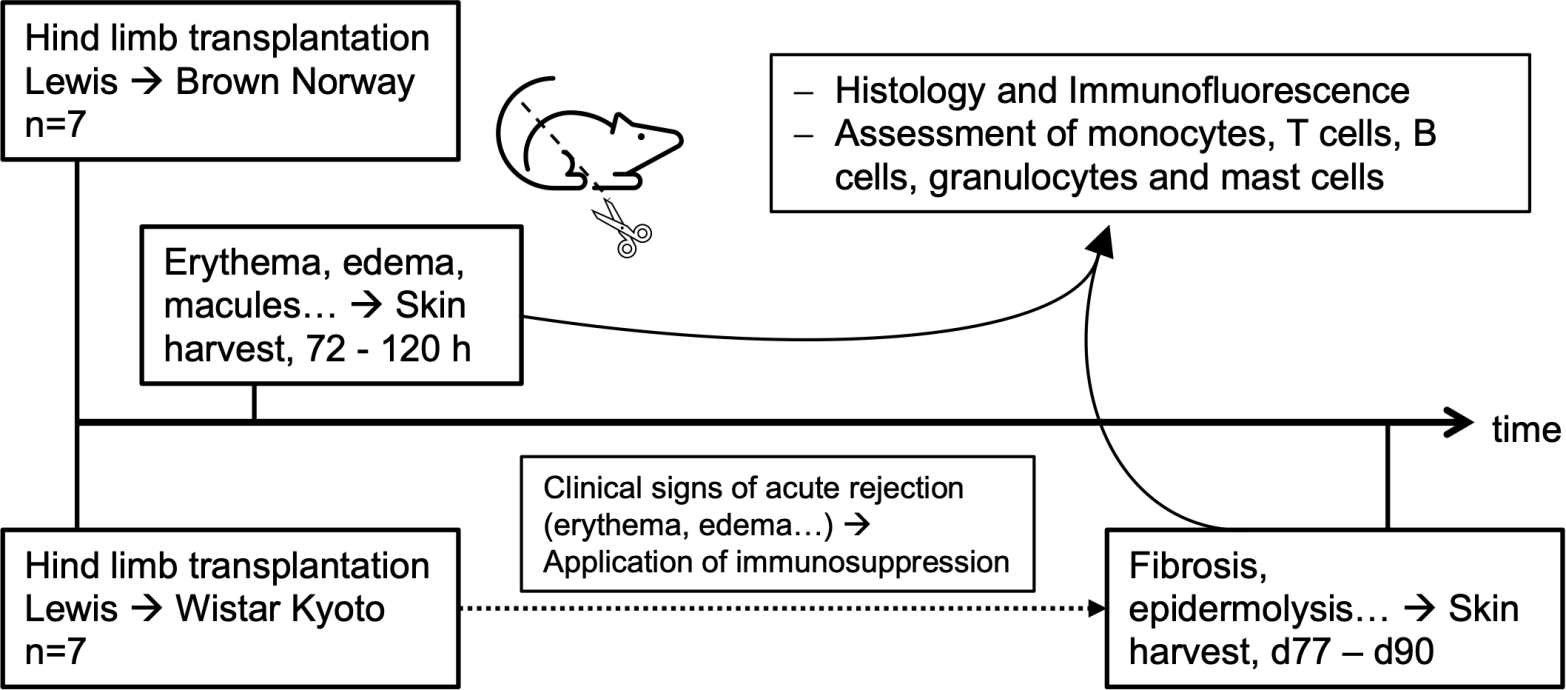
Study design. Timeline starts with orthotopic allogenic transplantation of Lewis hind limb on Brown Norway rat for acute and on Wistar Kyoto rat for chronic rejection. The procedures for acute rejection are shown above the time line, for chronic rejection below the timeline. Skin harvests were conducted when clinical signs of rejection emerged. For acute rejection this was after 72-120h, showing redness, edema, macules progressing to reddish-infiltrated lichenoid plaques and finally epidermolysis. For chronic rejection, harvest was conducted after 77–90 days, when fibrosis, loss of adnexa and epidermolysis were evident.

Study design is shown in **Figure 1**.

### 2.2 Sample preparation

For AR, the animals were euthanized in compliance with the German Animal Welfare Act after 72–120 hours. Clinical signs of rejection were defined as endpoint. The skin of both the healthy and the transplanted leg was carefully separated from the remaining tissue and preserved separately. The samples were then embedded in O.C.T.™ Tissue Tek^®^ and cryopreserved in liquid nitrogen. The tissue blocks were cryosectioned into consecutive 5 µm semithin sections and fixed in acetone within 30–60 minutes post-sectioning. The same procedure was followed for CR samples between 77 to 90 days post-transplantation, depending on chronic signs of rejection. Specimen adequacy was ensured through full-thickness skin samples comprising epidermis, dermis, adnexa, subcutaneous tissue, and vessels (23). All samples were stained with Hematoxylin Eosin (HE), Periodic Acid Schiff (PAS) staining and Masson’s Trichrome staining (MTC) for histopathological assessment as previously described (32, 33). Immunofluorescence was performed with monoclonal antibodies Anti-CD45R-PE (B cells), Anti-Granulocytes-PE (granulocytes), Anti-CD4-PE (T helper cells) and Anti-CD8 (Cytotoxic T cells) as well as Anti-CD68 (monocytes) with Anti-mouse-Alexa Fluor 594 as secondary antibody, as shown in **Supplementary Table 1**. Mast cells were addressed by Toluidine Blue staining. All samples were stained in triplicates and digitalized as a whole slide image using *Axioscan 7* (Carl Zeiss Microscopy, Oberkochen, Germany, 20x magnification). Cell counting was also performed on whole slide images in technical duplicates in a blinded fashion (Zeiss ZEN 3.3 Software, Oberkochen, Germany).

### 2.3 Pathologist’s assessment

**Figure 2:**
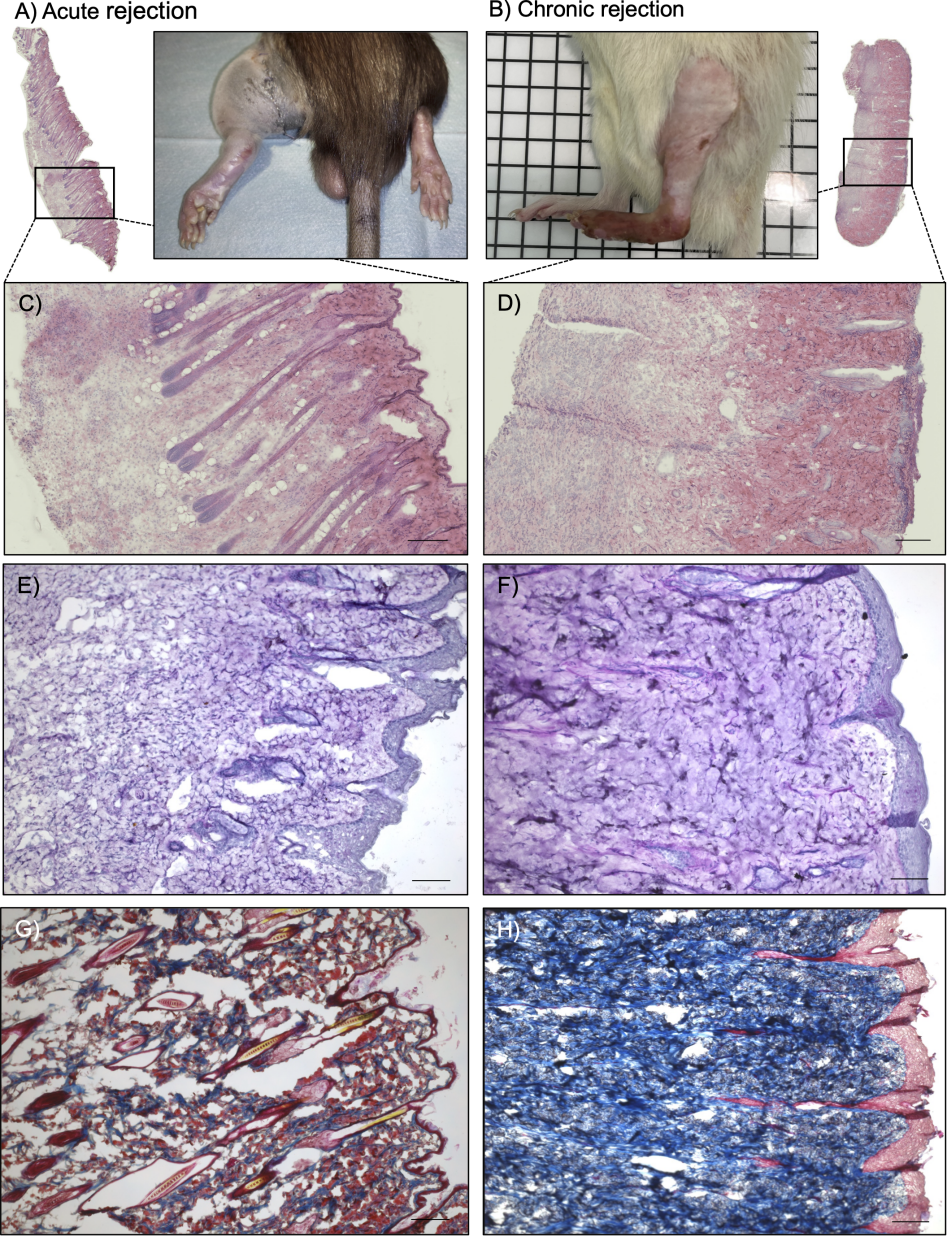
Histological and pathological assessment. Clinical pictures of rat hind limbs after 120 hours **(A)** and on day 90 **(B)**. Whole slide images were acquired for all samples. Hematoxylin Eosin Staining in acute **(C)** and chronic **(D)** rejection, scale bar = 200µm. Periodic Acid Schiff staining in acute **(E)** and chronic **(F)** rejection, scale bar = 100µm. Notice severe dermal edema and severe perivascular infiltrates in **(C, E)**, compared to vascular narrowing, loss of adnexa and again severe perivascular infiltrates in **(D, F)**. Masson’s trichrome staining in acute **(G)** and chronic **(H)** rejection. Collagen stained blue, indicating tissue fibrosis in chronic rejection. Scale bar = 100µm.

Histopathological assessment was manually performed on digitalized HE-, PAS- and MTC- stained whole tissue sections (**Figure 2**) of rodent skin using *HALO Image Analysis Platform* (Version 3.6.4134, Indica Labs, Albuquerque, New Mexico). Each sample was examined for signs of rejection in a blinded fashion by an experienced pathologist according to the Banff 2007 working classification of skin-containing composite tissue allograft pathology (23) also taking into account the current Banff VCA meeting report of 2022 (25). Typical morphological changes seen generally in chronic allograft rejection of solid organ transplants were adapted to the specific morphological features of VCA as observed in cases of CR and considered in analysis (34). Additionally, these criteria were homogenized with those named in the revised Banff scoring system (25). Defined criteria are shown in **Tables 1** and **2**. In addition to histopathological rejection criteria, the clinical appearance was visually assessed and used for confirmation of ongoing rejection (23), strengthening the translational relevance of this methodological approach.

**Table 1:** Criteria for acute rejection in accordance to the original Banff 2007 working classification of skin-containing composite tissue allograft pathology (23).

Criteria	Manifestation
Perivascular infiltrates	Rare, Mild, Moderate, Severe
Epidermal involvement	No, Spongiosis, Exocytosis, Spongiosis + Exocytosis
Adnexal involvement	No, Mild, Moderate, Severe
Epidermal dyskeratosis	No, Yes
Epithelial apoptosis	No, Mild, Moderate, Severe
Epithelial keratinolysis	No, Mild, Moderate, Severe
Frank necrosis	No, Yes
Cellular infiltrate composition	Lymphocyte dominated, Mixed
Eosinophils	Not elevated, Mild elevated
Overall infiltration pattern	Interstitial diffuse, Interface dermatitis, Perivascular dominated
Dermal edema	No, Mild, Moderate, Severe
Banff-Grade	0, I, II, III

**Table 2:** Additional criteria for chronic rejection and Banff update 2024 (25).

Criteria	Manifestation
Vascular narrowing	No, Yes
Loss of adnexa	No, Mild, Moderate, Severe
Skin atrophy	No, Mild, Moderate, Severe
Muscle atrophy	No, Mild, Moderate, Severe
Fibrosis of deep tissue	No, Mild, Moderate, Severe
Hyperkeratosis	No, Mild, Moderate, Severe
Keratolysis	No, Mild, Moderate, Severe
Basal cell hydrophic changes	No, Yes
Band like lymphohistiocytic infiltrate	No, Yes
Satellite cell necrosis	No, Yes
Loss of rete ridges	No, Yes
Capillary thrombosis	t0, t1
Vasculitis	v0, v1, v2
Allograft Vasculopathy	av0, av1, av2

### 2.4 Statistical analysis

Statistical analyses were performed using GraphPad Prism 7.01 Software. Data was analyzed for normality distribution with Shapiro-Wilk test. One-way ANOVA and Dunnett’s correction for multiple comparisons was performed. Fisher’s exact test was used for contingency analysis. P-values <0.05 were considered significant.

## 3 Results

### 3.1 Banff criteria are highly sensitive to rejection in rodents

**Figure 3:**
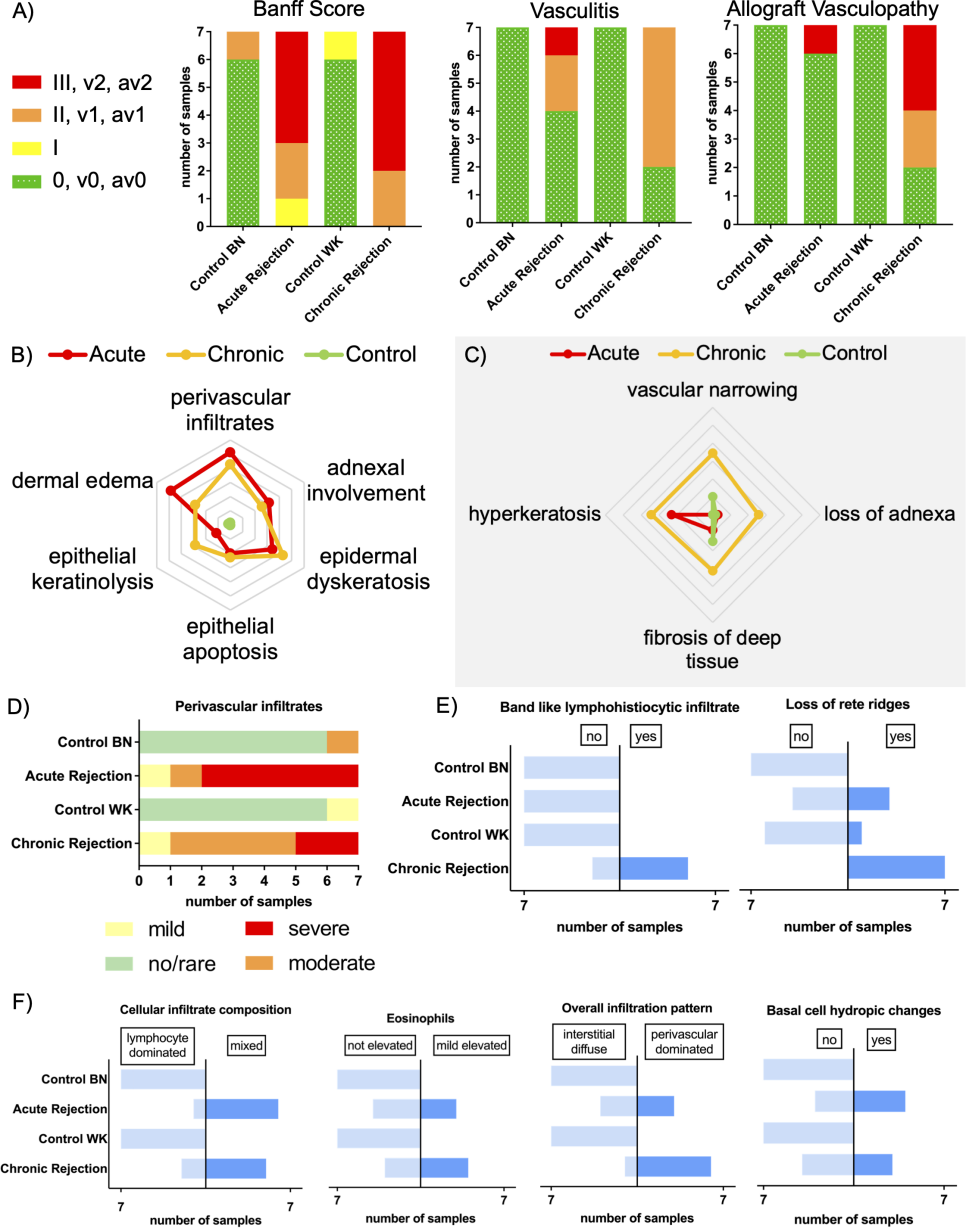
Comparison of acute and chronic rejection in terms of pathological criteria. **(A)** Number of samples in each Banff Score (0, I, II, III) with additional criteria of vasculopathy (v0, v1, v2) and allograft vasculopathy (av0, av1, av2). Histopathological criteria used for diagnosis of rejection as initially used **(B)** and recently updated **(C)** are indicated in the edges. Characteristics poorly (center) or strongly pronounced (edge). The lines represent the average of each group, consisting of n = 7 for AR and CR and n=14 for control. **(D)** Manifestation of perivascular infiltrates (no/rare, mild, moderate, severe) in different groups. Note similarity to Banff Score. **(E)** Manifestation of criteria as predominantly seen in chronic rejection. **(F)** Manifestation of criteria represented in both acute and chronic rejection. BN, Brown Norway; WK, Wistar-Kyoto.

All applied criteria appear to be highly sensitive to rejection in a rat hindlimb transplantation model. Almost no control samples exhibited features indicative of rejection as shown in **Figures 3B, C**, and hence were marked predominantly as Banff Score 0, v0, av0 (**Figure 3A**). Animals experiencing AR fulfilled significantly more frequent criteria for Banff Score (I, II, III) than healthy control skin (7/7 vs. 1/7, p=0.005, n=7). The same applies to animals experiencing CR (7/7 vs. 1/7, p=0.005, n=7). In each case, two animals reached Banff Score II (moderate rejection). In CR, five specimen fulfilled criteria for severe rejection (Banff III), whereas in AR four cases of severe rejection (Banff III) and one case of mild rejection (Banff I) were observed. The CR samples also presented significantly more often vasculitis (v1, v2, 5/7 vs. 0/7, p=0.02, n=7) and allograft vasculopathy (av1, av2, 5/7 vs. 0/7, p=0.02, n=7) when compared to healthy control skin. This was not the case for AR (vasculitis 4/7 vs. 0/7, p=0.19, n=7 and allograft vasculopathy 1/7 vs. 0/7, p>1.00, n=7). Capillary thrombosis was not found in examined samples.

Furthermore, clinical signs of AR were identified prior to histology in each case (27, 35): redness to erythema, edema, and macules progressing to reddish-infiltrated lichenoid plaques over time. In CR, the clinical appearance was mainly fibrotic with loss of adnexa and heightened susceptibility to skin injury (**Figures 2A, B**). These findings support the sensitivity of the Banff classification in preclinical settings, and underscore its potential as a standardized methodological framework for VCA research.

### 3.2 Histopathological criteria between acute and chronic rejection show a significant overlap

The majority of criteria for AR as suggested by Banff 2007 (23) were equally fulfilled by both AR and CR, whereas healthy control skin met nearly none of the criteria.

Criteria which are both applicable to AR and CR patterns are adnexal involvement, epidermal dyskeratosis, epithelial apoptosis or epithelial keratinolysis (**Figure 3B**). Epithelial keratinolysis appeared mild to moderate in three out of seven cases of AR, and severe in four out of seven cases in CR. Epidermal involvement in terms of spongiosis and/or exocytosis was observed in five cases each. In AR there were three cases with mildly elevated eosinophils and four cases with basal cell hydropic changes. CR showed similar results with 4 cases of mildly elevated eosinophils and three cases of basal cell hydropic changes (**Figure 3F**). In both AR and CR, the infiltration pattern of immune cells changed from lymphocyte dominated to mixed with the overall infiltration pattern changing from interstitial diffuse to perivascular dominated, which was most pronounced in CR (**Figure 3F**). Perivascular infiltrates were seen in both groups and further assessed by their severity. In AR, five samples showed severe perivascular infiltrates and one sample each mild or moderate perivascular infiltrates. Instead, in CR only 2 samples showed severe perivascular infiltrates, but five moderate and one mild (**Figure 3D**).

Regarding initial criteria of Banff 2007 working classification, only dermal edema seems to be exclusive for AR. Here, four samples of acute rejection showed severe edema, whereas no or mild edema was observed in four cases of chronic rejection.

### 3.3 Diagnosing chronic rejection requires specific criteria, including allograft vasculopathy

While acute and chronic rejection share certain features, there are identifiable criteria that distinguish them from one another. According to current literature, features of CR refer mainly to vascular changes, such as vascular narrowing, vasculitis/arteritis, capillary thrombosis and myointimal growth (26, 36–39). Vasculitis/arteritis is described as presence of mononuclear cells beneath endothelium (25).

Regarding the recent update of the Banff classification (25), this new criteria of vasculitis and allograft vasculopathy were predominantly met by samples of CR as described in **Figure 3A**.

As shown in **Figure 3C**, the manifestation of criteria for CR was more severe in specimen of CR than those of AR. Vascular narrowing was observed in five out of seven cases in CR but only one case of AR (5/7 vs. 1/7, p=0.10, n=7), as shown in **Supplementary Figure 1**. Three out of seven cases experiencing CR showed severe loss of adnexa, in AR there was only one sample with mild loss of adnexa. Fibrosis of deep tissue was seen more often in CR than in AR (6/7 vs. 2/7, p=0.10, n=7).

Band like lymphohistiocytic infiltrates were exclusively observable in CR, where five of seven samples met this criterion (5/7 vs. 0/7, p=0.02, n=7). Furthermore, loss of rete ridges was seen more often in samples with CR than in AR (7/7 vs. 3/7, p=0.07, n=7) as shown in **Figure 3E**.

Moreover, there were individual cases that should be mentioned. Satellite cell necrosis was observed in one case of CR and muscle atrophy in two cases of this group. Skin atrophy was seen in two cases of CR and in one specimen of AR. Additionally, in one rat experiencing AR, interface dermatitis was observed.

### 3.4 Immune cell infiltration of the skin characterizes acute and chronic rejection

**Figure 4:**
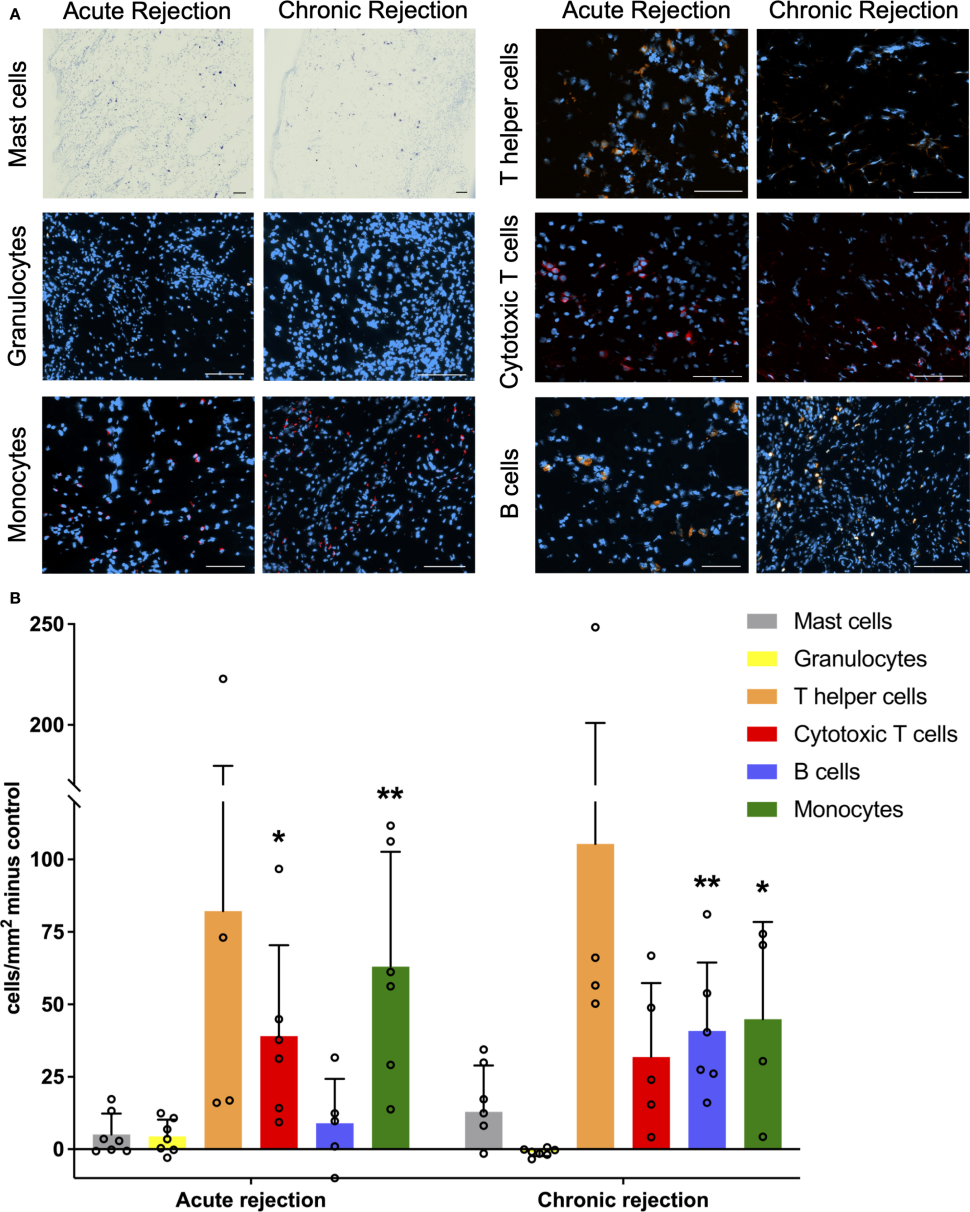
Immune cells in skin rejection. **(A)** Representative pictures of Toluidine Blue staining (Mast cells) and Immunofluorescence Anti-Granulocytes-PE, Anti-CD68-Alexa Fluor 594 (Monocytes), Anti-CD4-PE (T helper cells), Anti-CD8-Alexa Fluor 594 (Cytotoxic T cells) and Anti-CD45R-PE (B cells) for acute and chronic rejection respectively. Scale Bar = 100µm. **(B)** Number of cells per mm^2^ in transplantation skin minus corresponding healthy control leg. Statistical comparison against healthy legs. *p<0.05 and **p<0.01.

To investigate the impact of rejection on the infiltration of immune cells in the skin, values for the healthy contralateral leg were subtracted from those during transplant rejection, respectively. Mast cells, granulocytes, T helper cells, cytotoxic T cells, B cells and monocytes were assessed individually by immunofluorescence or Toluidine Blue staining (**Figure 4A**).

All of them showed increased infiltration setting control skin as a baseline, except for granulocytes in CR, which were mildly diminished (**Figure 4B**). For certain immune cells, significant individual variations have been observed, thereby accounting for the partially large standard deviation.

Monocytes significantly infiltrated the skin in both AR and CR (AR p=0.002, n=7, CR p=0.02, n=7) when compared to healthy control skin. In contrast, there was a significant infiltration of cytotoxic T cells (p=0.02, n=7) in AR compared to healthy control skin. This was not the case for CR (p=0.21). Although the infiltration by helper T cells is evident, statistical significance was not achieved neither in acute nor chronic rejection due to high individual differences. B cell infiltration increased in CR and was significantly different not only to healthy control skin (p=0.002, n=7) but also to AR (p=0.02, n=7). Granulocyte infiltration was significantly increased in AR in comparison to CR (p=0.02, n=7), but there was no difference to healthy control skin. Regarding immune cell infiltration, no other significant differences were observed between AR and CR. For mast cells there was no significant difference between any rejection type or healthy control skin nor between AR and CR.

## 4 Discussion

In this study, the Banff working classification was used in rodent VCA models to compare the application of the diagnostic criteria in acute and chronic rejection in the skin of allotransplants. Considerable overlap in the manifestation of histopathological criteria in AR and CR was observed. For diagnosing CR, specific criteria in terms of vascular alterations were used and able to distinguish from AR. Furthermore, band like lymphohistiocytic infiltrates and loss of rete ridges should be considered when diagnosing CR, whereas skin edema seems to be indicative of AR. Immune cells infiltrated the skin in both AR and CR.

Since the majority of VCA research is conducted in rat models, a uniform system for grading rejection should be used. The Banff classification, although initially arranged for human skin, serves as a valid grading system, as shown by this study. Though, there are several differences between the skin of rats and humans. For example, rat skin has additional layers, such as a thin muscle layer called Panniculus or the stratum fibrosum serving as the lower boundary of the subcutaneous tissue (40). Additionally, the skin on rat hindlimbs is usually significantly hairier than skin as part of VCAs, such as in hand or face allografts. This might even offer advantages when it comes to assessing changes in the adnexa. These observations not only validate the use of Banff criteria in rat models but also emphasize the need for ongoing methodological adaptation in experimental transplantation research.

Perivascular infiltrates are one crucial criterion in the histopathological diagnosis of rejection. Their manifestation aggravates with ongoing rejection and the severity of rejection correlates with the amount of involved vessels and perivascular lymphocytic infiltrates (23), which was confirmed by this study. This correlation between the extent of perivascular infiltrates and the Banff score becomes particularly evident when comparing **Figures 3A**, **D**. In this context perivascular infiltrates also often correlate with skin rash (41, 42). Although we were able to identify vasculitis as a significant criterion only in chronic rejection, we observed it in both AR and CR. Other studies have described lymphocytic vasculitis as an early sign of rejection in facial transplantation (43).

Looking at individual leukocyte subtypes, it becomes clear how crucial their contribution and collaboration is in working immune system. Mast cells participate in the interaction with regulatory T cells when it comes to allograft tolerance (15). Furthermore, they are capable of activating innate immunity (44). Monocytes were the only leukocyte subtype that showed significantly increased infiltration in both acute and chronic rejection, underlining their crucial role in allograft rejection (45), despite their occurrence in peripheral blood being limited to 2-8% of all leukocytes. Also, as confirmed by this study, cytotoxic T cells play a significant role in acute allograft rejection (12, 46). Interestingly, skin infiltrating T cells appear to be not only of recipient origin but deriving from the donor as well (12, 43). Recent studies by Kauke-Navarro et al. emphasize the potential of regulatory T cells as precision medicine for the future of VCA, showcasing the evolving landscape of immunomodulatory approaches (16). Even though the skin is presumed to be most exposed to allograft rejection, it also provides the opportunity for local immunosuppressive therapy (29, 47, 48). The impact of this therapeutic option on the characteristics presented in this study needs further investigation. Although consumption of neutrophils at the rejection site is suggested by previous studies (49), we did not see significant infiltration of granulocytes neither in AR nor CR.

Since the introduction of the first consensus scoring system for the rejection of vascularized composite allografts in 2007 (23), which primarily focused on AR, several cases of CR have been documented (28, 37, 50). As suggested in the latest update of the Banff classification, new data are required to validate the proposed changes (25). This study demonstrates that the implemented modifications were urgently necessary to systematically identify not only AR but also chronic alterations. Thus, this study serves as a test for the updated Banff classification and proves its validity.

Nevertheless, there are different types of CR, which were mainly described for solid organ transplantation so far. In kidney transplantation, CR is further divided into chronic active T cell mediated rejection and chronic active ABMR (51). 2019 Banff classification for kidney allograft rejection distinguishes between active ABMR, chronic active ABMR, chronic (inactive) ABMR and C4d staining without evidence for rejection (52). Although ABMR happens rarely in VCAs (10–13), similarities regarding classification of rejection types should be considered.

Some examples for human VCA allograft rejection were recently added to the literature. Krezdorn et al. described the coexistence of alterations in regards to acute and chronic rejection in the skin in two cases of face transplantation, speaking of chronic active rejection (53). In another face transplant acute rejection is described to smolder into chronic rejection (54), which might contribute to alterations like allograft vasculopathy. Although in our cohort allograft vasculopathy seemed to be an exclusive criterion for chronic rejection, other authors describe chronic skin alterations like sclerosis or fibrosis and loss of rete ridges, as also observed by us, as more consistent criteria for CR (28, 55). In contrast to solid organ transplantation, the development of vasculopathy in VCAs does not appear to rely exclusively on the presence of donor-specific antibodies, but exemplary on T-cell/macrophage–associated arteriosclerosis, which is frequently not captured in punch biopsies (54, 56). While diagnostic biopsies should remain as practical and minimally invasive as possible, the additional examination of deeper tissue samples may nevertheless be advantageous. In drawing conclusions about vasculopathy, it appears that some pathways involve affecting the vessel wall, whereas others do not.

It also remains unclear what influence the temporal component has on the development of chronic rejection. While some cases show no signs of chronic rejection even within 10 years, acute rejection episodes still occur during the same period (28). The most severe rejection episodes are observed within the first 12 months postoperatively (57, 58). In a longitudinal follow-up of six face transplants, only one patient did not experience any episode of AR before diagnosis of CR and all patients presented with severe signs of AR at the time of CR diagnosis (59). Such gradual transitions between AR and CR could potentially explain the simultaneous histologic presentation of acute and chronic changes in some of our samples as well, speaking also of chronic active rejection.

Cell-mediated rejection progresses through different phases, with various cell types appearing to be involved to differing degrees during early and late stages of rejection (58), while the timing of a biopsy reflects only a snapshot in time and does not capture the dynamic process. The administration of immunosuppressants can significantly reduce the occurrence of AR episodes in compliant patients (60, 61). Nonetheless, CR occurs over time, suggesting that despite the dominance of T cells, other cell types may be more relevant than initially assumed (58). This means that mechanisms are involved which are not adequately addressed or are untargeted by traditional immunosuppressive regimes or even triggered by multiple episodes of AR (26, 62).

There are some limitations in this study. First of all, a rodent model was used, and despite many parallels, its transferability to humans remains limited. Our model does not include mucosal tissue, instead, it contains the bony skeleton distal to the transfemoral coaptation and thereby most closely reflects hand transplantation. Grade 4 of the Banff classification was generally not attainable for animal welfare reasons, so the attribute of necrosis could not be observed. Moreover, the number of infiltrated immune cells in the skin does not necessarily provide information about their activation state. Although all criteria used are sensitive to rejection, they are not quite specific when considered on their own. Given the challenge of distinguishing rejection reactions from non-rejection pathologies such as infections (63), such events cannot be fully excluded, which might explain exceptions in healthy control groups. Also, we did not investigate on ischemia-reperfusion-injury in this study. One case of interface dermatitis may indicate signs of a particularly severe form of rejection or suggest an alternative cause for skin alterations (23).

Despite these limitations, this study is a successful comparison between acute and chronic rejection in skin of rodent VCA models based on the recently updated Banff working-classification of skin-containing composite tissue allograft pathology. Many criteria were fulfilled similarly by both AR and CR. At present, several questions regarding the distinction between AR and CR remain unanswered. There is no single criterion that can reliably differentiate chronic from acute rejection with absolute certainty. One reason may be the low specificity of individual criteria, as observed in this study. In addition, there is no uniform manifestation of CR; rather, multiple pathways appear to be involved, and numerous transitional forms exist. In our cohort, the most distinctive features of CR, in addition to allograft vasculopathy, were loss of rete ridges and band-like lymphohistiocytic infiltrates. We therefore advocate for an integrative diagnostic approach that considers the described criteria in conjunction with clinical presentation.

Ultimately, the etiology of chronic rejection still remains unclear. Is it a sign of repeated inflammation leading to permanent tissue changes and subsequently being overtaken by recurrent acute episodes of rejection? This would, at least in part, explain the significant overlap between criteria for AR and CR. Since certain criteria like vascular changes or loss of rete ridges are almost exclusively observed in CR, we suggest their occurrence as a specific consequence of chronic inflammation in the pathology of CR. We advise future studies to further focus on the underlying mechanisms, alongside the continued development and validation of standardized methods for diagnosing and monitoring chronic rejection in VCA.

## Data Availability

The original contributions presented in the study are included in the article/**Supplementary Material**, further inquiries can be directed to the corresponding author.
