# Supplementary material for: Differentiation of acute versus chronic skin rejection in a rodent model of vascularized composite allotransplantation
Source: Front Immunol. 2025 Sep 30;16:1672754. doi: 10.3389/fimmu.2025.1672754 (PMC12518069; doi:10.3389/fimmu.2025.1672754)
Supplement: Supplementary file 1 [file DataSheet1.pdf]

Supplementary Material

Supplementary Figure 1)

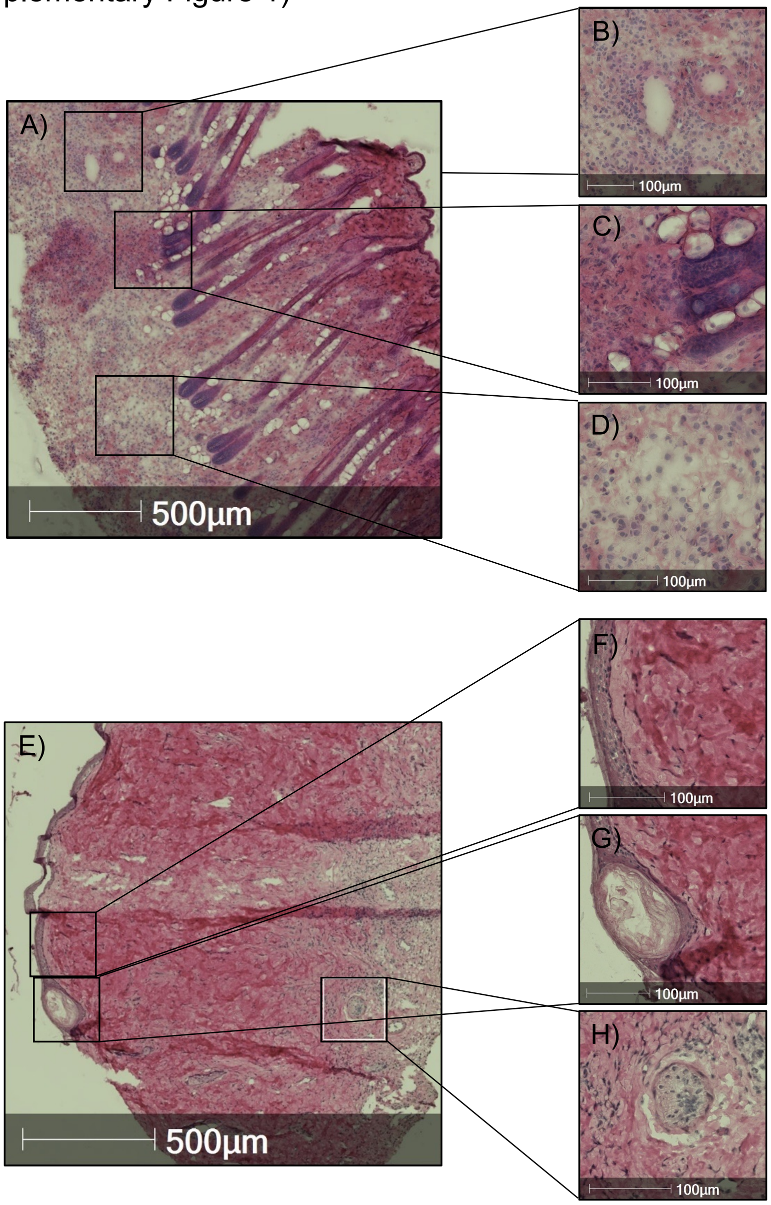

Supplementary Figure 1: (A) Illustration of acute rejection in accordance to the original Banff 2007 working classification on H.E.-slides (2X). Notice the severe perivascular immune cell infiltration (B, 10X), involvement of adnexal structures (C, 10X) and dermal edema (D, 10X) in higher magnification.

(E) Illustration of chronic rejection in accordance to additional criteria for chronic rejection and Banff Update 2024 on H.E.-slides (2X). Notice massive collagenous fibrosis, loss of rete ridges, loss of adnexa and skin atrophy (F, 10X) as well as hyperkeratosis (G, 10X) and vascular narrowing (H, 10X) in higher magnification.

Supplementary Table 1: Additional information on antibodies used for immunofluorescence.

Name	Dilution	Supplier	Catalogue number	Clone	RRID
Anti-CD4-PE	1:100	Thermo Fisher Scientfic, Waltham, USA	12-0040-82	OX35	RRID:AB_2572548
Anti-CD8	1:50	Cedarlane Laboratories, Burlington, Canada	CL004AP	OX-8	RRID:AB_10059877
Anti-CD68	1:100	Millipore Sigma, Burlington, USA	MAB1435	ED-1	RRID:AB_177576
Anti-CD45R-PE	1:100	Thermo Fisher Scientific, Waltham, USA	12-0460	His24	RRID:AB_465692
Anti-Granulocytes-PE	1:100	Santa Cruz, Biotechnology, Dallas, USA	Sc-19613	His48	RRID:AB_627686
Goat anti-mouse IgG Alexa Fluor 594	1:500	Thermo Fisher Scientific, Waltham, USA	A-11032		RRID:AB_2534091
